# Occurrence of *Mycobacterium avium *subspecies *paratuberculosis *across host species and European countries with evidence for transmission between wildlife and domestic ruminants

**DOI:** 10.1186/1471-2180-9-212

**Published:** 2009-10-07

**Authors:** Karen Stevenson, Julio Alvarez, Douwe Bakker, Franck Biet, Lucia de Juan, Susan Denham, Zoi Dimareli, Karen Dohmann, Gerald F Gerlach, Ian Heron, Marketa Kopecna, Linda May, Ivo Pavlik, J Michael Sharp, Virginie C Thibault, Peter Willemsen, Ruth N Zadoks, Alastair Greig

**Affiliations:** 1 Moredun Research Institute, Pentlands Science Park, Bush Loan, Penicuik EH26 0PZ, UK; 2 Centro de Vigilancia Sanitaria Veterinaria (VISAVET), Universidad Complutense, Avenida Puerta de Hierro s/n, 28040 Madrid, Spain; 3 Central Institute of Wageningen University, Edelhertweg 15, 8200 AB Lelystad, The Netherlands; 4 UR1282, Infectiologie Animale, Santé Publique (IASP-311), INRA centre de Tours, F-37380 Nouzilly, France; 5 Departamento de Sanidad Animal, Facultad de Veterinaria, Universidad Complutense, Avenida Puerta de Hierro s/n, 28040 Madrid, Spain; 6 Veterinary Institute of Thessaloniki, NAGREF, Thermi 57001, P.B.O: 60272 Thessaloniki, Greece; 7 Institut für Mikrobiologie Stiftung Tierärztliche Hochschule Hannover, Bischofsholer Damm 15, 30173 Hannover, Germany; 8 Veterinary Research Institute, Hudcova 70, 621 00 Brno, Czech Republic; 9 Veterinary Laboratories Agency, Pentlands Science Park, Bush Loan, Penicuik EH26 0PZ, UK; 10 Scottish Agricultural College, Veterinary Science Division, Cleeve Gardens, Oakbank Road, Perth, UK; 11 Laboratoire Microorganismes, Génomes et Environnement, UMR CNRS 6023, Université Blaise Pascal, 63177 Aubière cedex, France

## Abstract

**Background:**

*Mycobacterium avium *subspecies *paratuberculosis *(*Map*) causes an infectious chronic enteritis (paratuberculosis or Johne's disease) principally of ruminants. The epidemiology of *Map *is poorly understood, particularly with respect to the role of wildlife reservoirs and the controversial issue of zoonotic potential (Crohn's disease). Genotypic discrimination of *Map *isolates is pivotal to descriptive epidemiology and resolving these issues. This study was undertaken to determine the genetic diversity of *Map*, enhance our understanding of the host range and distribution and assess the potential for interspecies transmission.

**Results:**

164 *Map *isolates from seven European countries representing 19 different host species were genotyped by standardized IS*900 *- restriction fragment length polymorphism (IS*900*-RFLP), pulsed-field gel electrophoresis (PFGE), amplified fragment length polymorphisms (AFLP) and mycobacterial interspersed repeat unit-variable number tandem repeat (MIRU-VNTR) analyses. Six PstI and 17 BstEII IS*900*-RFLP, 31 multiplex [SnaBI-SpeI] PFGE profiles and 23 MIRU-VNTR profiles were detected. AFLP gave insufficient discrimination of isolates for meaningful genetic analysis. Point estimates for Simpson's index of diversity calculated for the individual typing techniques were in the range of 0.636 to 0.664 but a combination of all three methods increased the discriminating power to 0.879, sufficient for investigating transmission dynamics. Two predominant strain types were detected across Europe with all three typing techniques. Evidence for interspecies transmission between wildlife and domestic ruminants on the same property was demonstrated in four cases, between wildlife species on the same property in two cases and between different species of domestic livestock on one property.

**Conclusion:**

The results of this study showed that it is necessary to use multiple genotyping techniques targeting different sources of genetic variation to obtain the level of discrimination necessary to investigate transmission dynamics and trace the source of *Map *infections. Furthermore, the combination of genotyping techniques may depend on the geographical location of the population to be tested. Identical genotypes were obtained from *Map *isolated from different host species co-habiting on the same property strongly suggesting that interspecies transmission occurs. Interspecies transmission of *Map *between wildlife species and domestic livestock on the same property provides further evidence to support a role for wildlife reservoirs of infection.

## Background

*Mycobacterium avium *subspecies *paratuberculosis *(*Map*) causes paratuberculosis or Johne's disease, a fatal chronic granulomatous enteritis. The disease occurs worldwide and is responsible for significant economic losses to livestock and associated industries [1,2]. It is endemic in Europe with only Sweden maintaining paratuberculosis-free status. The epidemiology is poorly understood and there are important questions still to resolve, particularly with respect to interspecies transmission. *Map *infects principally ruminants but over the past decade it has become apparent that the organism has a much broader host range including monogastric species [3-5]. The infection of humans with *Map *and possible association with Crohn's disease remains a controversial issue and requires more study [6,7]. The strain types involved and the extent to which interspecies transmission occurs have still to be elucidated. Evidence also is accumulating regarding the existence of potential wildlife reservoirs, for example, infected rabbits appear to be a particular problem in some areas of Scotland [3] but the role of such wildlife reservoirs in the epidemiology of the disease has still to be clarified.

Our knowledge and understanding of the epidemiology of *Map *has been hindered for many years by our inability to discriminate *Map *from the environmental species of *Mycobacterium avium *(*M. avium*) and to differentiate between *Map *isolates from different host species and different geographic locations. Recent advances in molecular biology have led to the refinement and development of molecular typing methods with sufficient discriminatory power to differentiate between *M. avium *subspecies and different *Map *isolates [8]. Genome analyses have revealed two major strain groups designated 'Type I', or 'sheep or S type' and 'Type II' or 'cattle or C type'. A sub-type of Type I strains designated 'Type III' or 'intermediate or I type' is found in sheep and goats. All three of these strain types can be differentiated by restriction fragment length polymorphism coupled with hybridization to IS*900 *(IS*900*-RFLP) [9,10] or pulsed-field gel electrophoresis (PFGE) analyses [11,12] and by a PCR assay based on single nucleotide polymorphisms in the *gyrA *and *gyrB *genes [13]. Single nucleotide polymorphisms in the IS*1311 *element also distinguish three types designated 'S' (sheep), 'C' (cattle) and 'B' (bison) [14,15]. In this case the assay cannot distinguish between Types I and III and the 'B' type is a sub-type of Type II strains. *In silico *genome comparisons and techniques such as representational difference analysis and microarray analysis have identified sequence polymorphisms unique to either Type I or II strains and these have been used to develop PCRs for discriminating these strain groups [16-21]. The purpose of this study was to investigate the molecular diversity of *Map *isolates from a variety of hosts across Europe to enhance our understanding of the host range and distribution of the organisms and assess the potential for interspecies transmission. Previous studies have revealed limited genetic diversity; therefore, to maximise strain differentiation we evaluated several different molecular typing techniques in isolation and in combination; IS*900*-RFLP, PFGE and PCR-based techniques including amplified fragment length polymorphisms (AFLP) and mycobacterial interspersed repeat unit-variable number tandem repeat (MIRU-VNTR).

## Results

AFLP typing was performed at the Central Institute of Wageningen University, Lelystad, The Netherlands and MIRU-VNTR at INRA, Nouzilly, France. For PFGE and IS*900*-RFLP typing, the field isolates were split between two labs. PFGE typing was undertaken at the Moredun Research Institute, Scotland, UK and VISAVET, Madrid, Spain. IS*900*-RFLP typing was carried out at the Veterinary Research Institute in Brno, Czech Republic and VISAVET. Published standardized typing procedures were used as described in Materials and Methods. The only difference in procedures between laboratories was that at VISAVET the IS*900*-RFLP analysis was performed using the agarose plugs prepared for PFGE to avoid having to perform two separate DNA preparations for the different typing techniques. The correct profiles were reported by all laboratories for the duplicate isolates included to check reproducibility. All typing techniques correctly reported that the *Mycobacterium phlei (M. phlei)*, *Mycobacterium bovis *BCG (*M. bovis *BCG) and IS*901 *positive *M. avium *were not *Map*. One field isolate, EU112 was found to be IS*901 *positive *M. avium *(it is not known if the isolate is *M. avium *subsp. *avium *or *M. avium *subsp. *silvaticum*) and not *Map *as was originally suspected. Another isolate, EU169 was found to be a mixed culture. Isolates one to 50 were typed at Institut für Mikrobiologie Stiftung Tierärztliche Hochschule Hannover, Hannover, Germany using the Type I/Type II PCR as described by Dohmann *et al*. [17]. EU25 and EU30 were identified as Type I and all other field isolates as Type II. These results correlated with the strain type as determined by PFGE. This PCR [17] cannot discriminate between Type I and Type III and as strain types could be discerned from the PFGE profiles, it was not considered necessary to determine the strain type of the remaining isolates by PCR. It was not possible to type all of the isolates with all typing methods as some laboratories had difficulties in subculturing some isolates to prepare sufficient cells for analyses. A total of 123 *Map *isolates were typed by IS*900*-RFLP, PFGE and MIRU-VNTR.

### IS*900*-RFLP typing

IS*900*-RFLP typing data were obtained for 147 *Map *isolates (Table 1 and see supplementary dataset in Additional file 1). It was not possible to obtain PstI profiles for 55 isolates or clear BstEII profiles for five isolates. There was a problem using agarose plug DNA for IS*900*-RFLP typing with PstI as the enzyme would not cleave in the presence of agarose. Extraction of the DNA from the agarose and repeat PstI digestion was not attempted. As expected, profiles were not obtained for the negative control strains *M. bovis *BCG, *M. phlei *and IS*901 *positive *M. avium*. A total of six PstI profiles were found among 93 isolates: B (n = 88); G (n = 1); I (n = 1); K (n = 1); R (n = 1); and U (n = 1). Seventeen BstEII profiles were detected among 142 isolates: C1 (n = 71); C17 (n = 49); C5 (n = 5); C9 (n = 3); C16 (n = 2) and single isolates with C10, C18, C22, C27, C29, C35, C36, C38, C39, S4, I4 and I5. Ten different combined PstI-BstEII profiles were recorded among the 88 isolates that were characterised with both enzymes: B-C1 (n = 42); B-C17 (n = 36); B-C9 (n = 3) and single isolates of B-C5, B-C16, G-C35, I-C29, K-C17, R-I4 and U-C16. The B-C17 profile was predominant in Scotland in this cohort of isolates, specifically in the regions of Aberdeenshire, Angus, Borders and Perth and Kinross (Table 1 and see supplementary dataset in Additional file 1 and Additional file 2: Table S1). The C1 profile was more widely spread across Europe and was found in the Czech Republic, Greece, Finland, The Netherlands, Norway and Spain, (Table 1 and see supplementary dataset in Additional file 1 and Additional file 2: Table S1).

**Table 1:** Combined PFGE, MIRU-VNTR and IS*900*-RFLP profiles by *Map *origin

Profile			No of isolates	Country^1^-Host^2^						
**PFGE**^3^	**MIRU-VNTR**^4^	**IS900-RFLP**^5^		**CZ**	**ES**	**FL**	**GR**	**NL**	**NO**	**SCO**

[1-1]	1	C1	2	RD				G		
[1-1]	2	C1	7	C, RD	C	C(2)		C, RD		
[1-1]	2	C18	1			C				
[1-1]	2	C5	1					C		
[1-1]	6	C1	2					C(2)		
[2-1]	1	C1	13	C(4), FD, M	C	C(2)			G(3), S	
[2-1]	1	C9	1	H						
[2-1]	1	C17	39						C, S	B, C(6), CR, F(2), H, R(13), RK, S(7), ST(3), W, WM
[2-1]	2	C17	2							C(2)
[2-1]	2	C1	9	C	FD			C(2), G, S(4)		
[2-1]	2	C5	1					C		
[2-1]	2	C36	1					C		
[2-1]	5	C10	1	C						
[2-1]	19	C17	1							S
[2-1]	24	C1	1					S		
[2-1]	22	C38	1					G		
[2-1]	25	C17	1							R
[2-10]	1	C1	1						G	
[2-17]	2	C22	1					S		
[2-19]	2	C5	2				G, S			
[2-30]	1	C16	1					RD		
[2-30]	25	C16	1							W
[3-2]	1	C17	3							F, G, J
[5-2]	1	C17	1							S
[9-7]	21	S4	1							S
[15-16]	38	C1	1		G					
[15-25]	26	C1	7		G(7)					
[16-11]	20	I5	1		G					
[18-1]	13	C1	1		G					
[20-1]	1	C1	1	C						
[26-1]	35	C1	1	C						
[27-18]	2	C27	1		C					
[29-15]	36	C1	1				G			
[29-15]	37	C1	3				G(3)			
[30-21]	2	C1	1					G		
[31-17]	69	C39	1		G					
[32-29]	1	C17	1							ST
[34-22]	2	C1	2					RD(2)		
[34-22]	8	C1	1					RD		
[36-27]	1	C1	1	M						
[37-23]	29	I4	1	FD						
[40-28]	26	C1	1		G					
[41-1]	1	C9	1	C						
[58-64]	35	C1	1	M						

^1. ^Country: CZ Czech Republic, ES Spain, FL Finland, GR Greece, NL The Netherlands, NO Norway, SCO Scotland

^2. ^Host: B badger (*Meles meles*), C cow (*Bos taurus*), CR crow (*Corvus corone*), F fox (*Vulpes vulpes*), FD fallow deer (*Dama dama*), G goat (*Capra hircus*), H hare (*Lepus europaeus*), J jackdaw (*Corvus monedula*), M moufflon (*Ovis musimon*), R rabbit (*Oryctolagus cuniculus*), RD red deer (*Cervus elaphus*), RK rook (*Corvus frugilegus*), S sheep (*Ovis aries*), ST stoat (*Mustela erminea*), W weasel (*Mustela nivalis*), WM wood mouse (*Apodemus sylvaticus*). The number of isolates obtained from each host species within a country is given in parenthesis.

^3. ^Nomenclature as defined by Stevenson *et al*. 2002 [11]

^4. ^Nomenclature as defined by Thibault *et al*. 2007 [56]

^5. ^Nomenclature as defined by Pavlik *et al*. 1999 [50]

### PFGE typing

**Figure 1:**
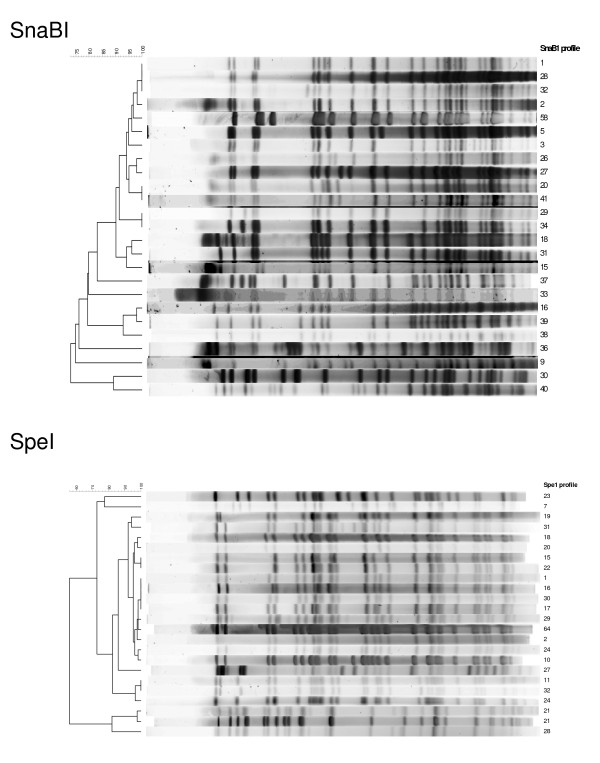
**Dendrograms showing the genetic relationships between the SnaBI and SpeI PFGE profiles of the *Map *isolates analysed in the study**. The similarity coefficients were calculated using Dice and hierarchical cluster analysis of the data was performed using the unweighted pair group method with arithmetic means.

PFGE typing data were obtained for 145 *Map *isolates (Table 1 and Figure 1). Twenty four different SnaBI profiles were detected in this panel of isolates: 2 (n = 91); 1 (n = 15); 15 (n = 9); 29 (n = 4); 34 (n = 4); 3 (n = 3); 38 (n = 2) and 5, 9, 16, 18, 20, 26, 27, 30, 31, 32, 33, 36, 37, 39, 40, 41, 58 (n = 1 each); and 23 distinct SpeI profiles: 1 (n = 102); 25 (n = 8); 2, 15, 22 (n = 4 each); 17, 19, 21, 30, 32 (n = 2 each) and 7, 10, 11, 16, 18, 20, 23, 24, 27, 28, 29, 31, 64 (n = 1 each). The combination of both enzyme profiles gave 31 different multiplex profiles: [2-1] (n = 83); [1-1] (n = 15); [15-25] (n = 8); [29-15],[34-22] (n = 4 each); [3-2] (n = 3); [2-19],[2-30],[38-32] (n = 2 each) and [2-10], [2-17], [2-21], [2-31], [5-2], [9-7], [15-16], [16-11], [18-1], [20-1], [26-1], [27-18], [30-21], [31-17], [32-29], [33-20], [36-27], [37-23], [39-24], [40-28], [41-1],[58-64] (n = 1 each). By far the most widely distributed PFGE type was [2-1], which was found in the Czech Republic, Finland, The Netherlands, Norway, Scotland and Spain (Table 1 and see supplementary dataset in Additional file 1 and Additional file 2: Table S1). PFGE type [1-1] was the next most common occurring in the Czech Republic, Finland, The Netherlands and Spain (Table 1 and see supplementary dataset in Additional file 1 and Additional file 2: Table S1). Profile [2-30] was found in The Netherlands and Scotland and the other profiles were found in only one country (Table 1 and see supplementary dataset in Additional file 1 and Additional file 2: Table S1). The numbers of isolates detected with these profiles are too small to determine if these multiplex profiles truly are restricted in their geographical location.

### AFLP typing

A representative subset of 68 *Map *isolates in the typing panel were analysed by AFLP. The DNA restriction patterns generated by EcoRI and MseI showed patterns that met the conditions for analyses such as fragment sizes, number of bands and ratio of fully versus partially digested fragments. The *Map *isolates, as a group, clearly clustered differently from other mycobacterial species such as *Mycobacterium marinum, Mycobacterium tuberculosis *and *M. phlei*. However, within the group of *Map *isolates a low degree of genetic diversity was detected, with isolates displaying between 90 and 95% homology. The reproducibility of the technique was assessed and it was concluded that on average the calculated similarities using the Pearson product-moment correlation between AFLP typing repeats was 85 to 90%. Since the variation detected between repeat analyses was in the same range as the genetic variation detected between *Map *isolates it was concluded that AFLP could not discriminate effectively between isolates and no further *Map *isolates were typed using this procedure.

### MIRU-VNTR typing

One hundred and forty seven *Map *isolates were typed by MIRU-VNTR and 23 different types were obtained (Table 1 and see supplementary dataset in Additional file 1). In addition, MIRU-VNTR types INMV 23 and 28 were obtained for the two IS*901 *positive *M. avium *isolates. The following MIRU-VNTR types were exhibited by *Map *isolates in this study: INMV 1 (n = 75); 2 (n = 35); 26 (n = 9); 6 (n = 4), 37 (n = 3), 8, 25, 35 (n = 2); 5, 13, 19, 20, 21, 22, 24, 27, 29, 30, 31, 32, 36, 38, 69 (n = 1). INMV 1 and 2 were the most widely disseminated MIRU-VNTR types, both occurring in the Czech Republic, Finland, The Netherlands, Scotland and Spain (Table 1 and see supplementary dataset in Additional file 1 and Additional file 2: Table S1). INMV 1 also was found in Norway and INMV 2 in Greece (Table 1 and see supplementary dataset in Additional file 1 and Additional file 2: Table S1). The relative frequencies of the various alleles were calculated and are shown in Table 2. The allelic diversity observed is consistent with the previous report [22].

**Table 2:** MIRU-VNTR Allelic diversity among the *Map *isolates.

**No. of isolates with specified MIRU copy No**.												
Locus	0	1	2	3	4	5	6	7	8	9	10	Allelic diversity (h)
292			14	47	80	3	2		1			0.58
10		21	126									0.24
7		10	128	9								0.22
3	10	6	131									0.2
25		2		138		7						0.1
X3		6	139	2								0.09
47			1	142	4							0.06
32									146	1		0.006

The allelic diversity (***h***) at a locus was calculated as ***h ***= 1 - Σ***x***_*i*_^2 ^[***n***/(***n ***- 1)], where ***x***_*i *_is the frequency of the ***i***th allele at the locus, and ***n ***the number of isolates [52,53].

### Comparison of typing techniques

A predominance of one or two types was observed with all of the typing techniques and these predominant types could be further discriminated by one or both of the other typing methods (Table 3). For example, the predominant PFGE multiplex type [2-1] comprising 83 isolates was subdivided into nine different profiles by MIRU-VNTR and seven different profiles by BstEII IS*900*-RFLP. PFGE multiplex type [1-1] comprising 15 isolates could be subdivided into three INMV profiles and three BstEII IS*900*-RFLP patterns. Minor multiplex profiles [2-30], [29-15] and [34-22]  were each subdivided into two by MIRU-VNTR. The major MIRU-VNTR type INMV1 consisting of 75 isolates was split by PFGE into 11 and by BstEII IS*900*-RFLP into four subtypes. INMV2 composed of 35 isolates was subdivided into eight and seven types by PFGE and BstEII IS*900*-RFLP, respectively. The minor groups INMV 6, 8, 25, 26 and 35 were each subdivided by PFGE into a further two types and INMV 25 into two BstEII types. Both PFGE and MIRU-VNTR each differentiated the most widespread BstEII IS*900*-RFLP type C1, which included 71 isolates, into 14 and 11 distinct types, respectively. The BstEII type C17 comprising 49 isolates was subdivided into four types by each of the other typing methods. The minor types C5 and C9 were further subdivided into three and two, respectively, by PFGE and VNTR-MIRU subdivided C16 into two types. By combining PFGE and VNTR-MIRU or all three typing techniques it was possible to discriminate 37 and 44 patterns, respectively (Table 4 and see Additional file 2: Table S2).

**Table 3:** Subdivision of the predominant types by the different typing techniques.

Type	**No. of isolates**^1^	Subdivided by		
		
		**BstEII RFLP**^2^	**PFGE**^3^	**MIRU-VNTR**^4^
[2-1]	83	C1, C5, C9, C10, C17, C36, C38		1, 2, 5, 8, 19, 22, 24, 25, 30
[1-1]	15	C1, C5, C18		1, 2, 6
[29-15]	4	C1		36, 37
[34-22]	4	C1		2, 8
[2-30]	2	C16		25, 1
INMV 1	75	C1, C9, C16, C17	[1-1], [2-1], [2-10], [2-30], [3-2], [5-2], [20-1], [32-29], [33-20], [36-27], [41-1]	
INMV 2	35	C1, C5, C17, C18, C22, C27, C36	[1-1], [2-1], [2-17], [2-19], [2-31], [27-18], [30-21], [34-22]	
INMV 26	9	C1	[15-25], [40-28]	
INMV 6	4	C1	[1-1], [2-21]	
INMV 25	2	C16, C17	[2-1], [2-30]	
INMV 8	2	C1	[2-1], [34-22]	
INMV 35	2	C1	[26-1], [58-64]	
C1	71		[1-1], [2-1], [2-10], [15-16], [15-25], [18-1], [20-1], [26-1], [29-15], [30-21], [34-22], [36-27], [40-28], [58-64]	1, 2, 6, 8, 13, 24, 26, 35, 36, 37, 38
C17	49		[2-1], [3-2], [5-2], [32-29]	1, 2, 19, 25
C5	5		[1-1], [2-1], [2-19]	2
C9	3		[2-1], [41-1]	1
C16	2		[2-30]	1, 25

^1. ^123 *Map *isolates were typed by IS*900*-RFLP, PFGE and MIRU-VNTR but not all isolates were typed by all three typing procedures.

^2. ^Nomenclature as defined by Pavlik *et al*. 1999 [50]

^3. ^Nomenclature as defined by Stevenson *et al*. (2002) [11]

^4. ^INMV numbers as defined by INRA Nouzilly MIRU-VNTR [56]

**Table 4:** Simpson's index of diversity (SID) with 95% confidence interval for individual and combined typing methods

	All isolates		Scotland		Mainland Europe	
**Method**	**No. of types**	**SID**	**No. of types**	**SID**	**No. of types**	**SID**

PFGE-SnaBI	21	0.594 (0.493-0.695)^a^	5	0.234 (0.075-0.393)^ab^	17	0.744 (0.655-0.834)^ac^
PFGE-SpeI	19	0.485 (0.372-0.597)^a^	5	0.267 (0.105-0.430)^ab^	16	0.599 (0.468-0.729)^ab^
PFGE-multiplex	26	0.654 (0.558-0.749)^ab^	6	0.270 (0.104-0.437)^ab^	22	0.804 (0.727-0.881)^acd^
IS*900*-RFLP	15	0.636 (0.582-0.690)^a^	3	0.080 (0.00-0.191)^a^	14	0.422 (0.277-0.567)^b^
MIRU-VNTR	19	0.664 (0.588-0.740)^ab^	5	0.235 (0.074-0.395)^ab^	16	0.770 (0.706-0.835)^ac^
Multiplex PFGE + IS*900*-RFLP	34	0.834 (0.782-0.885)^c^	6	0.270 (0.104-0.437)^ab^	30	0.877 (0.82-0.934)^cde^
Multiplex PFGE + MIRU-VNTR	37	0.797 (0.727-0.867)^bc^	9	0.406 (0.228-0.584)^ab^	30	0.914 (0.878-0.949)^de^
IS*900*-RFLP + MIRU-VNTR	29	0.825 (0.774-0.876)^c^	6	0.236 (0.074-0.398)^ab^	24	0.868 (0.820-0.917)^cde^
All methods combined	44	0.879 (0.831-0.927)^c^	9	0.406 (0.228-0.584)^b^	36	0.941 (0.913-0.969)^e^

Simpson's index of diversity (SID) with 95% confidence interval for individual and combined typing methods based on analysis of 123 *Map *isolates originating from Scotland (n = 48) and mainland Europe (n = 75) ^abcde ^Non-overlapping 95% confidence intervals are considered significantly different [55] and are indicated by different superscripts.

### Genetic diversity

Simpson's Index of Diversity (SID) with 95% confidence intervals for the individual typing techniques and their combinations based on the analysis of 123 *Map *isolates for which results were obtained by the IS*900*-RFLP, PFGE and MIRU-VNTR methods are given in Table 4. SID values are given for the combined European dataset (all isolates), for the Scottish isolates and for the isolates from mainland Europe. When comparing SIDs, differences were considered statistically significant when there were no overlaps between the confidence intervals. The phylogenetic relationships between the isolates are shown in Figure 1 using PFGE data.

### Distribution among different host species

*Map *isolates from three domestic species of ruminants and 14 different wildlife species, a feral cat and a captive giraffe were typed (Table 1 and see supplementary dataset in Additional file 1 and Additional file 2: Table S3). The wildlife encompassed both ruminant and non-ruminant species. Among the wildlife species, feral cat and captive giraffe, a total of nine IS*900*-RFLP, nine PFGE and six INMV types were detected.

In order to make a preliminary assessment of transmission dynamics, the combined typing data from all three molecular techniques was considered, as this was most discriminatory. A total of seven combined profiles were detected in isolates from more than one host species ([1-1], INMV1, C1; [1-1], INMV2, C1; [2-1], INMV1, C1; [2-1], INMV1, C17; [2-1], INMV2, C1; [2-19], INMV2, C5; [3-2], INMV1, C17) (Table 1). The evidence for interspecies transmission is more compelling if the same strain types are isolated from multiple species on the same property. Even with the limited data available on the properties from which the isolates in the study were obtained, it was possible to show that two combined profiles ([2-1], INMV1, C17 and ([2-19], INMV2, C5) were found in more than one species on the same property in seven cases (Table 5). Of these, four properties included isolates from both livestock and wildlife (EN, DR, I and R). The properties CF, DR and I, are all located within the geographical area of Perth and Kinross and EN, GE and R in the adjacent region of Angus in Scotland. Isolates from species on all six of these properties had the same combined profile ([2-1], INMV1, C17). Profile [2-19], INMV2, C5 was obtained from a goat and a sheep on the same property in Greece.

**Table 5:** *Map *strain types infecting multiple host species on a single property

Property	Typing profile	Species
EN	[2-1] INMV1 C17	Cow, hare, rabbit, rook, stoat
CF	[2-1] INMV1 C17	Crow, fox, rabbit (5)
DR	[2-1] INMV1 C17	Cow, rabbit (4), woodmouse
GE	[2-1] INMV1 C17	Fox, stoat (2), weasel
I	[2-1] INMV1 C17	Rabbit, sheep
R	[2-1] INMV1 C17	Cow, rabbit
KV	[2-19] INMV2 C5	Goat, sheep

Numbers in parenthesis indicate the number of animals of that species identified with the given typing profile

Limited data was available for two properties in the Czech Republic, KRH and VO. On these properties the combined typing profiles of the isolates showed that they were not the same in all the species sampled. The PFGE multiplex profile [2-1] was found on VO in isolates from both a cow and a hare but IS*900*-RFLP analysis showed the hare isolate to have a different profile to the cow. The two deer on property KRH had a different profile to that of a cow on the same farm.

## Discussion

The results of this study improve our knowledge of the epidemiology of paratuberculosis in Europe regarding the genetic diversity and distribution of *Map *isolates with respect to geographic location and host species of origin. The study has also permitted a comprehensive comparison of three standardized typing procedures, the results of which will inform future epidemiological studies as to the most appropriate and discriminative methods to employ.

This is the first study to compare the discriminatory power of IS*900*-RFLP, PFGE, AFLP and MIRU-VNTR for the molecular characterization of *Map *isolates. AFLP could not effectively discriminate between *Map *isolates and therefore is not suitable for epidemiological studies on paratuberculosis. A major problem with the technique was reproducibility. This was probably due in part to the variable quality of the mycobacterial DNA, which is highly dependent on growth phase and difficult to extract from *Map *isolates that are particularly resilient to lysis. Reproducibility could also have been affected by small variations in the experimental procedure such as shifts in electrophoretic mobility during capillary electrophoresis. Despite several attempts using alternative analytical procedures, no decrease in this variation could be obtained.

The most widely used measure of diversity is Simpson's Index of Diversity (SID), which we have employed here to estimate the discriminatory power of the various molecular typing techniques utilised in this study. When all *Map *isolates were considered irrespective of host or geographic origin, the SID was not significantly different between each of the individual typing techniques (IS*900*-RFLP, multiplex PFGE and MIRU-VNTR) and was low at a value between 0.636 and 0.664 in accordance with previous reports [23,24]. The SID value is strongly influenced by the distribution of types rather than the number of types detected. This is clearly demonstrated by comparing the two methods with the largest difference in the number of patterns detected i.e. IS*900*-RFLP, which identified 15 profiles and multiplex PFGE, which detected 26 profiles. Despite the number of profiles detected, both methods have almost the same SID point estimate and 95% confidence interval. The SID for IS*900*-RFLP could have been improved further had it been possible to obtain PstI profiles for the isolates. The discriminatory power of the individual techniques is too low for epidemiological surveys since a SID of around 0.9 is generally considered the minimum. For isolates from mainland Europe, SID for the combination of multiplex PFGE and MIRU-VNTR, with or without IS*900*-RFLP, exceeded the threshold value of 0.9. The increase in SID is not surprising since the different typing techniques target different sources of genetic variation and have different limitations and will therefore complement each other when used in combination. Due to limited heterogeneity among Scottish isolates, combining all three typing techniques increased SID to 0.879 for the dataset as a whole, providing discriminatory power close to the minimum but not quite reaching the target value.

Although the combination of all three typing techniques gives the greatest discrimination, this is generally not practical or cost effective for large national or international studies and often a compromise is sought. The choice of typing method will be influenced by the predominant isolate type in the population to be tested. This is highlighted in this study by considering the data shown in Table 4 for the isolates from Scotland versus those from mainland Europe and the combined European dataset (i.e. all isolates). The isolates from Scotland comprise a homogeneous population in which the B-C17 IS*900*-RFLP profile predominates and is therefore a rigorous test for the combination approach. Comparing the SIDs for the various combinations of typing techniques there was no difference between multiplex PFGE + MIRU-VNTR and the combination of all three typing techniques. Therefore, a combination of multiplex PFGE + MIRU-VNTR would be suitable for epidemiological studies in Scotland. A combination of multiplex PFGE + MIRU-VNTR would also be appropriate for mainland Europe but here a combination of IS*900*-RFLP and multiplex PFGE would also perform well. The best combination for the combined European dataset was all three typing techniques. The SID for the isolates from mainland Europe was often higher than that for the combined European dataset, the latter being affected by the inclusion of the less heterogeneous Scottish isolates. Based on these results a small pilot study of the population of interest is recommended before undertaking a large epidemiological survey. For further epidemiological studies in Scotland, it would be advantageous to undertake a pilot study including short sequence repeat analysis [25], which may improve the discriminatory power for this homogeneous population of isolates.

The study identified the common isolate types within the European countries examined. IS*900*-RFLP profile C1 was the most widespread, consistent with previous reports from individual countries [26-31]. This profile has a global distribution, being found in the United States, Australia and New Zealand [10,30,32]. Although IS*900*-RFLP profile C17 is commonly isolated in Scotland it is reported to be relatively rare in other European countries [30,31]. It was identified in isolates from The Netherlands and Norway in this study and has been reported previously in Germany [31] and is predominant in specific regions of Argentina [30,33]. The most common PFGE profile was [2-1] found in six of the seven countries examined, closely followed by [1-1] found in four. INVM 2 was found in six countries and INVM 1 in five. Further investigations will be required to determine if this distribution is a consequence of animal movements, increased virulence or whether these isolates have characteristics that allow them to transmit more readily. There is evidence to suggest that different mycobacterial strain types vary in their ability to cause disease. Caws *et al*. [34] provided evidence that *M. tuberculosis *genotype influences clinical disease phenotype and demonstrated a significant interaction between host and bacterial genotypes and the development of tuberculosis. Gollnick *et al*. [35] reported that the survival of *Map *in bovine monocyte-derived macrophages was not affected by host infection status but by the infecting strain type. Two recent studies suggest that different *Map *strain types may play a role in polarizing the host immune responses during infection [36,37]. Also, different *Map *strains have been found to differ in virulence in experimental infections of deer [38] and in a mouse model (KS, unpublished data) and Verna *et al*. have provided data to show how the strain type may influence the pathology of ovine paratuberculosis [39].

Surprisingly, no Type I strains (corresponding to S Type strains in the literature [40]) were identified within the 27 sheep and 33 goat field isolates submitted by the partners. This may be a reflection of the difficulties encountered in isolating and growing these strains *in vitro*. Typically, isolates of strain Type I are slow-growing, taking longer than 16 weeks and sometimes as long as 18 months to isolate on solid medium. Cultures are often not retained this long in diagnostic laboratories. Furthermore, studies have shown that the decontamination procedures or media used for isolation can significantly affect recovery of these strains. Reddacliff *et al*. [41] reported the detrimental effects of various decontamination protocols on the recovery of Type I strains from tissues and faeces. The addition of egg yolk and mycobactin J to BACTEC 12B or 7H9 broth was found to be essential for the isolation of Australian sheep strains from faeces and to enhance their recovery from tissue samples [42]. Other workers have successfully isolated Type I or III strains on LJ or Middlebrook 7H11 supplemented with mycobactin J [43,44]. The addition of antibiotics can also affect growth. Both ampicillin and vancomycin hydrochloride can retard growth of Type I strains [45]. The various laboratories participating in this study used a range of decontamination procedures and culture media but it is not possible to rule out a culture bias.

The results of this survey highlight an interesting difference between the epidemiology of *Map *in Europe and Australia. This study shows that in Europe, Type II strains (corresponding to C Type strains in the literature [10]) are commonly isolated from sheep, goats and cattle whereas in Australia, Type II strains are rarely, if ever, isolated from sheep -the predominant type being Type I. We can only speculate as to the reasons for this difference. Management practices will affect the circulation of strains and can differ between some parts of Europe and Australia. The scale of farming operations and relative proportions of the different livestock co- or sequentially grazing may also be a factor. Paratuberculosis is more common in sheep in Australia than in cattle and the Type I strain is more virulent for sheep than cattle [39].

In this study, *Map *was isolated from 19 different host species, which included both ruminants and non-ruminants. This is the first report of the isolation of *Map *from a giraffe. The Type II strains appear to have greater propensity for infecting a broad range of host species whereas the epidemiological data available for Type I strains suggests that they have a preference for sheep and goats [23]. Since our results show that the same profiles are found in isolates from different species, it strongly suggests that strain sharing occurs. Even more convincing was the observation that the same profiles were isolated from wildlife species and domestic ruminants on the same farm. The frequency or ease with which interspecies transmission occurs are unknown entities and require further investigation. Similarly, the relative risk of transmission from domestic livestock to wildlife or vice versa remains to be determined.

All animals in contact with *Map *contaminated faeces on an infected property will contribute to the spread of disease through passive transmission. However, *Map *infects a variety of wildlife host species that potentially could be reservoirs for infection of domestic livestock and have serious implications for control of paratuberculosis. The role of wildlife reservoirs in the epidemiology of paratuberculosis will depend on a number of factors which need to be taken into consideration when undertaking a risk assessment for interspecies transmission. Although *Map *can infect many wildlife species, only wild ruminants and lagomorphs show evidence of disease as determined by the presence of gross or microscopic lesions with associated acid fast bacteria [46]. These wildlife species have the capacity to excrete *Map *and spread disease to other susceptible species primarily through further faecal contamination of the environment. Potentially, they could constitute wildlife reservoirs. By definition, to constitute a wildlife reservoir the infection would need to be sustained within the species population. Evidence is available for vertical, pseudovertical and horizontal transmission within natural rabbit populations which could contribute to the maintenance of *Map *infections within such populations [47,48].

The other wildlife species in this study could be categorised into predators and scavengers that probably acquire the disease through eating contaminated prey or carrion, respectively. It has been reported previously that these animals show no clinical signs of disease and only minor histopathological changes with a few acid fast bacteria in tissues [4,5]. Such infected predators and scavengers are probably 'dead-end hosts' and are not high risk factors for interspecies transmission.

Information pertaining to strain types can assist in designing and evaluating disease control programmes. It is beneficial to know the predominant strain type in a population or the virulence of a particular strain type particularly for developing new vaccines. Singh *et al*. [49] recently reported the effectiveness and advantage of using a vaccine based on a local 'bison-type' strain.

## Conclusion

In conclusion, this survey has helped to expand our knowledge to improve our understanding of the epidemiology of paratuberculosis. It is hoped that the information provided will facilitate future surveys and research strategies to resolve the outstanding epidemiological questions regarding this disease.

The results of this study were in agreement with previous reports indicating that *Map *isolates comprise a relatively homogeneous population exhibiting little genetic diversity compared with other bacterial pathogens. As a result it is necessary to use multiple genotyping techniques targeting different sources of genetic variation to obtain the level of discrimination necessary to investigate transmission dynamics and trace the source of infections. Identical genotypes were obtained from *Map *isolated from different host species co-habiting on the same property strongly suggesting that interspecies transmission occurs. Interspecies transmission of *Map *between wildlife species and domestic livestock on the same farm provides further evidence to support a role for wildlife reservoirs of infection. However, in assessing the relative risk of transmission between wildlife and domestic livestock, distinction needs to be made between passive and active transmission as well as the potential for contact.

## Methods

### Bacteria

A total of 166 suspected *Map *isolates were obtained from the Czech Republic (n = 27), Finland (n = 5), Greece (n = 6), The Netherlands (n = 46), Norway (n = 7), Scotland (n = 54) and Spain (n = 21) (Table 1 and see supplementary dataset in Additional file 1). The isolates from livestock species were obtained from animals showing symptoms of paratuberculosis and from various clinical samples (see supplementary dataset in Additional file 1) that were submitted to the various laboratories for diagnosis. In the case of isolates from wildlife species, these were isolated from wildlife on properties with a known history or current problem with paratuberculosis and these animals did not necessarily show any clinical signs. The isolates were cultured from 19 different host species (supplementary dataset in Additional file 1 and Additional file 2: Table S3). Isolates were propagated on slopes of one of the following media depending on what was used routinely in the supply laboratories:- modified Middlebrook 7H11 supplemented with 20% (vol/vol) heat-inactivated newborn calf serum, 2.5% (vol/vol) glycerol, 2 mM asparagine, 10% (vol/vol) Middlebrook oleic acid-albumin-dextrose-catalase (OADC) enrichment medium (Becton Dickinson, Oxford, Oxfordshire, United Kingdom), Selectatabs (code MS 24; MAST Laboratories Ltd., Merseyside, United Kingdom), and 2 μg ml^-1 ^mycobactin J (Allied Monitor, Fayette, Mo.); Herrold's egg yolk medium with 2 μg ml^-1 ^mycobactin J or Lowenstein-Jensen medium with 2 μg ml^-1 ^mycobactin J. For the typing panel, three *Map *isolates were included to represent the three strain types described in *Map *[11,12]. In addition, three isolates (one bovine, one ovine and one caprine) were duplicated in the panel as internal controls for the reproducibility of the typing methods and *M. bovis *BCG, *M. phlei *and IS*901 *positive *M. avium *(it is not known if this isolate is *M. avium *subsp. *avium *or *M. avium *subsp. *silvaticum*) were included as negative controls. The isolates were coded with an EU reference number (see supplementary dataset in Additional file 1) and genotyped in a blind study.

### IS*900*-RFLP method

The typing laboratories were provided either with cultures or with DNA in agarose plugs that had been prepared for PFGE typing. DNA extraction from cultures and IS*900*-RFLP analysis was performed using the standardized procedure published by Pavlik *et al*. [50]. Where plugs were provided, the restriction digests were carried out in the presence of agarose as described for PFGE [51]. Briefly, a 3-5 mm insert of agarose was cut from the plug, washed extensively in TE buffer and pre-incubated with the appropriate restriction buffer containing 0.1 mg ml^-1 ^BSA. After one hr the buffer was discarded and replaced with fresh buffer containing the restriction endonuclease and incubated overnight at 37°C. The agarose containing the digested DNA was then loaded into the wells of an agarose gel as described in the standardized procedure [51]. New profiles were designations assigned by the National Veterinary Institute, Brno using the standard nomenclature described. Profiles were analysed using Gel Compar (Biomathematics, Belgium).

### PFGE analysis

PFGE analysis was carried out using SnaBI and SpeI according to the published standardized procedure of Stevenson *et al*. [11] with the following modifications. Plugs were prepared to give a density of 1.2 × 10^10 ^cells ml^-1 ^and the incubation time in lysis buffer was increased to 48 hr. The concentration of lysozyme was increased to 4 mg ml^-1^. Incubation with proteinase K was carried out for a total of seven days and the enzyme was refreshed after four days. Restriction endonuclease digestion of plug DNA by SpeI was performed with 10 U overnight in the appropriate restriction endonuclease buffer supplemented with 0.1 mg ml^-1 ^BSA, after which the enzyme was refreshed and incubated for a further 6 hr. The parameters for electrophoresis of SpeI restriction fragments were changed to separate fragments of between 20 and 250 Kb as determined by the CHEF MAPPER and electrophoresis was performed for 40 hr. The modified conditions are available on the website [51]. Gel images were captured using an AlphaImager 2200 (Alpha Innotech). Profiles were analysed using Bionumerics Maths™ software (Applied Maths, Belgium).

### AFLP analysis

A loop of cells from a culture tube was resuspended in 1 ml H_2_O. The optical density was adjusted to 1 McFarland unit in order to standardize the performance of the subsequent DNA extraction. DNA was extracted using Instagene Matrix (Bio-Rad™) according to the manufacturer's instructions.

100 ng template DNA was digested for 2 hr with 1 unit EcoRI and MseI at 37°C. The 10 μl mixture contained: 5 μl template DNA, 1.0 μl (10×) BSA, 1.0 μl NEB 2 buffer, 0.05 μl EcoRI, 0.1 μl Mse I (NEB) and H_2_O and was incubated for 2 hr at 37°C.

Eco-adaptor (50 pmol μl^-1^), annealed from primer pair: 5'-ctcgtagactgcgtacc-3' and 5'-aattggtacgcagtctac-3'and Mse-adaptor (5 pmol μl^-1^) annealed from primer pair: 5'-gacgatgagtcctgag-3'and 5'-tactcaggactcatc-3' were ligated to the digested DNA by adding 5 μl of the ligation mixture (0.6 μl Eco-adaptor, 0.6 μl Mse-adaptor, 0.3 μl T4-ligase (NEB, 1 unit), 1.5 μl 5 M NaCl, 1.5 μl ligase buffer (10×) (NEB) and 0.5 μl H_2_O) to 10 μl of the RE-digestion mixture, followed by 2 hr incubation at 16°C.

The amplification reaction was carried out in a 10 μl mixture containing 5.0 μl DNA from the adaptor-ligation reaction, 1.2 μl H_2_O, 0.2 μl dNTP (10 mM), 1.0 μl PCR buffer (10× PCR buffer II, ABI), 0.6 μl MgCl_2 _(25 mM), 1.2 μl Mse-0 primer (50 ng μl^-1^) and 0.2 μl Amplitaq Taq polymerase (5 U). The PCR cycling conditions were: hold 2 min 72°C, 12 cycles: (30 sec, 65°C touch down 0.7 C per cycle, 60 sec 72°C), 23 cycles: (30 sec, 56 C, 60 sec, 72°C), 60 sec, 72°C, hold 4°C.

The PCR product was run on a capillary automated sequencer (ABI 3100 avant). The AFLP profiles were analysed with the Bionumerics software programme (Applied Maths).

### MIRU-VNTR analysis

DNA in agarose plugs prepared for PFGE analysis was used for MIRU-VNTR analysis. Small pieces of agarose plug, approximately 2 mm thick, were washed in TE buffer (pH 8) to remove residual EDTA in the storage buffer. One hundred microlitres of TE buffer were added to the agarose and the sample boiled for 10 min to melt the agarose and denature the DNA. Five microlitres (80 ng) were used for PCR and the MIRU-VNTR analysis was performed as described by Thibault *et al*. [22] detecting eight polymorphic loci. The allelic diversity (*h*) at a locus was calculated as *h *= 1 - Σ*x*_*i*_^2 ^[*n*/(*n *- 1)], where *x*_*i *_is the frequency of the *i*th allele at the locus, and *n *the number of isolates [52,53].

### Strain type analysis by PCR

Isolates were typed to differentiate between strain types I or II using the PCR reported by Dohmann *et al*. (2003)[17].

### Calculation of the discriminatory power

Simpson's index of diversity (SID) described by Hunter and Gaston [54] was used as a numerical index for the discriminatory power of PFGE, IS*900*-RFLP and VNTR and combinations of these typing methods. The SID was calculated using the data from 123 isolates that were typed with all three typing procedures using the following formula:

Where *N *is the total number of isolates in the typing scheme, *s *is the total number of distinct patterns discriminated by each typing method and strategy, and *n*_*j *_is the number of isolates belonging to the *j*th pattern. Confidence intervals of 95% were calculated according to Grundmann *et al*. [55].

## Authors' contributions

KS conceived of the study, participated in its design and coordination, collated and analysed the data and drafted the manuscript. JA, SD, ZD, KD, IH, LDJ, MK, LM, IP, VT, PW participated in the laboratory and field work. FB, IH, PW and RZ participated in analyzing the data. GFG, DB, JMS, AG participated in the conception, design and coordination of the study. All authors read, criticized and approved the final manuscript.

## Supplementary Material

Additional file 1 **Complete dataset**. Complete dataset with information on host species of origin, clinical sample used for isolation, geographical location and typing data for individual isolates included in the study. Click here for file

Additional file 2 Supplementary tables listing the genotypes obtained with the combined typing techniques of IS*900*-RFLP, PFGE and MIRU-VNTR and documenting the distribution of *Map *molecular types according to geographical location and host species. Click here for file
