# Supplementary material for: Occurrence of Mycobacterium avium subspecies paratuberculosis across host species and European countries with evidence for transmission between wildlife and domestic ruminants
Source: BMC Microbiol. 2009 Oct 7;9:212. doi: 10.1186/1471-2180-9-212 (PMC2765967; doi:10.1186/1471-2180-9-212)
Supplement: Additional file 2 — Supplementary tables listing the genotypes obtained with the combined typing techniques of IS900-RFLP, PFGE and MIRU-VNTR and documenting the distribution of Map molecular types according to geographical location and host species. [file 1471-2180-9-212-S2.PDF]

Supplementary Table 1: Geographical distribution of *Map* molecular types

Country	No. of isolates[a]	IS*900*-RFLP profiles[b]	PFGE multiplex profiles[c]	MIRU-VNTR types (INMV Nos)[d]
Czech Republic	27	B-C1, B-C9, RI4, C10	[1-1], [2-1], [20-1], [26-1], [36-27], [37-23], [41-1], [58-64]	1, 2, 5, 29, 35
Finland	5	C1, C18	[1-1], [2-1]	1, 2
Greece	6	C1, C5	[2-19], [29-15]	2, 36, 37
Netherlands	45	B-C1, B-C5, C22, G-C35, C36, C38, I-C29, K-C17, U-C16	[1-1], [2-1], [2-17], [2-21], [2-30], [2-31], [30-21], [34-22]	1, 2, 6, 8, 22, 24, 30
Norway	7	C1, C17	[2-1], [2-10]	1
Spain	21	B-C1, C27, C39, I5	[1-1], [2-1], [15-16], [15-25], [16-11], [18-1], [27-18], [31-17], [38-32], [39-24], [40-28]	1, 2, 13, 20, 26, 27, 31, 32, 38, 69
Scotland	54	B-C16, B-C17	[2-1], [2-30], [3-2], [5-2], [9-7], [32-29], [33-20]	1, 2, 19, 21, 25

[a]Total number of isolates from each country. Note that not all isolates were typed by each typing method.
[b] Nomenclature as defined by Pavlik *et al*. 1999 [50]. PstI profiles not obtained for some isolates.
[c]Nomenclature as defined by Stevenson *et al* (2002) [11]
[d]INMV numbers as defined by Thibault *et al*., (2007) [22]

Supplementary Table 2: Combination of PFGE, MIRU-VNTR and IS*900* RFLP typing methods

PFGE[a] + MIRU-VNTR[b]	No. of isolates	PFGE[a] + MIRU-VNTR[b] + IS*900* RFLP[c]	No. of *Map* isolates
[1-1],1	2	[1-1],1,C1	2
[1-1],2	9	[1-1],2,C1	7
[1-1],6	2	[1-1],2,C18	1
[2-1],1	53	[1-1],2,C5	1
[2-1],2	13	[1-1],6,C1	2
[2-1],5	1	[2-1],1,C1	13
[2-1],19	1	[2-1],1,C9	1
[2-1],22	1	[2-1],1,C17	39
[2-1],25	1	[2-1],2,C17	2
[2-1],24	1	[2-1],2,C1	9
[2-10],1	1	[2-1],2,C5	1
[2-17],2	1	[2-1],2,C36	1
[2-19],2	2	[2-1],5,C10	1
[2-30],1	1	[2-1],19,C17	1
[2-30],25	1	[2-1],24,C1	1
[3-2],1	3	[2-1],22,C38	1
[5-2],1	1	[2-1],25,C17	1
[9-7],21	1	[2-10],1,C1	1
[15-16],38	1	[2-17],2,C22	1
[15-25],26	7	[2-19],2,C5	2
[16-11],20	1	[2-30],1,C16	1
[18-1], 13	1	[2-30],25,C16	1
[20-1],1	1	[3-2],1,C17	3
[26-1],35	1	[5-2],1,C17	1
[27-18],2	1	[9-7],21,S4	1
[29-15],36	1	[15-16],38,C1	1
[29-15],37	3	[15-25],26,C1	7
[30-21],2	1	[16-11],20,I5	1
[31-17],69	1	[18-1],13,C1	1
[32-29],1	1	[20-1],1,C1	1
[34,22],2	2	[26-1],35,C1	1
[34-22],8	1	[27-18],2,C27	1
[36-27],1	1	[29-15],36,C1	1
[37-23],29	1	[29-15],37,C1	3
[40-28],26	1	[30-21],2,C1	1
[41-1],1	1	[31-17],69,C39	1
[58-64],35	1	[32-29],1,C17	1
		[34-22],2,C1	2
		[34-22],8,C1	1
		[36-27],1,C1	1
		[37-23],29,I4	1
		[40-28],26,C1	1
		[41-1],1,C9	1
		[58-64],35,C1	1

[a]Nomenclature as defined by Stevenson *et al* (2002) [11]
[b]INMV numbers as defined by INRA Nouzilly MIRU-VNTR [22]
[c] Nomenclature as defined by Pavlik *et al*. 1999 [50]

Supplementary Table 3: Distribution of *Map* molecular types among host species

Common name	Species name	No. of isolates	BstEII IS*900* RFLP profiles[a]	PFGE multiplex profiles[b]	MIRU-VNTR types (INMV Nos)[c]
Cow	*Bos taurus*	52	B-C1, B-C5, B-C17, C18	[1-1], [2-1], [2-21], [2-31], [20-1], [26-1], [27-18], [33-20], [38-32], [39-24], [41-1]	1, 2, 5, 6, 27, 30, 31, 32, 35
Sheep	*Ovis aries*	26	B-C1, B-C17, C5, C22, S4	[2-1], [2-17], [2-19], [5-2], [9-7]	1, 2, 19, 21, 24
Goat	*Capra hircus*	32	B-C1, I-C29, I5, C5, C17, C38, C39	[1-1], [2-1], [2-19], [2-10], [3-2], [15-16], [15-25], [16-11], [18-1], [29-15], [30-21], [31-17], [40-28]	1, 2, 8, 13, 20, 22, 26, 28, 36, 37, 38, 69
Moufflon	*Ovis musimon*	7	B-C1	[2-1], [36-27], [58-64]	1, 35
Red deer	*Cervus elaphus*	10	B-C1, K-C17, U-C16	[1-1], [2-1], [2-30], [34-22]	1, 2, 8
Fallow deer	*Dama dama*	4	B-C1, R-I4	[2-1], [37-23]	1, 2, 29
Badger	*Meles meles*	1	B-C17	[2-1]	1
Fox	*Vulpes vulpes*	3	B-C17	[2-1], [3-2]	1
Stoat	*Mustela erminea*	4	B-C17	[2-1], [32-29]	1
Weasel	*Mustela nivalis*	2	B-C16, B-C17	[2-1], [2-30]	1, 25
Crow	*Corvus corone*	1	B-C17	[2-1]	1
Rook	*Corvus frugilegus*	1	C17	[2-1]	1
Jackdaw	*Corvus monedula*	1	B-C17	[3-2]	1
Rabbit	*Oryctolagus cuniculus*	14	B-C17	[2-1]	1, 25
Hare	*Lepus europaeus*	2	B-C17, B-C9	[2-1]	1
Rat	*Rattus norvegicus*	1		[2-1]	1
Wood mouse	*Apodemus sylvaticus*	1	B-C17	[2-1]	1
Giraffe	*Giraffa camelopardalis*	1	C5		
Cat	*Felis domesticus*	1	G-C35		

[a] Nomenclature as defined by Pavlik *et al*. 1999 [50]. PstI profiles not obtained for some isolates.
[b]Nomenclature as defined by Stevenson *et al* (2002) [11]
[c]INMV numbers as defined by INRA Nouzilly MIRU-VNTR [22]
